# Association analysis of 374T/A (rs1800624) receptor for advanced glycation end-products (RAGE) gene polymorphism with diabetic retinopathy in Pakistani patients

**DOI:** 10.12669/pjms.37.3.3670

**Published:** 2021

**Authors:** Shazia Qayyum, Muhammad Afzal, Abdul Khaliq Naveed

**Affiliations:** 1 Dr. Shazia Qayyum, MPhil. Department of Pathology, Riphah International University, Islamabad, Pakistan; 2 Muhammad Afzal, MPhil. Department of Biochemistry, Riphah International University, Islamabad, Pakistan; 3 Prof. Dr. Abdul Khaliq Naveed, FCPS, PhD. Department of Biochemistry, Riphah International University, Islamabad, Pakistan

**Keywords:** Type-2 diabetes mellitus, Diabetic retinopathy, 374T/A RAGE Gene polymorphism, Soluble RAGE

## Abstract

**Objectives:**

to determine the relationship of 374T/A (rs1800624) polymorphism in the gene encoding RAGE with Type-2 diabetes mellitus (T2DM), diabetic retinopathy (DR) and serum soluble RAGE (sRAGE) level in Pakistani patients.

**Methods:**

A case-control study, conducted from January 2017 to December 2018, involving 150 healthy controls (HC), 150 T2DM patients with no retinopathy (DNR) and 150 DR patients diagnosed by coloured fundus photography. Tetra-primer amplification refractory mutation system – polymerase chain reaction (T-ARMS-PCR) was used for genotyping. Serum sRAGE levels were measured by enzyme-linked immunosorbent assays (ELIZA).

**Results:**

The frequency of TT, TA and AA genotypes of rs1800624 polymorphism were: 92.7%, 6%, 1.3% in HC, 80%, 17.3%, 2.7% in DNR and 76.7%, 19.3%, 4.3% in DR groups. Heterozygous TA genotype and mutant A allele showed significant association with diabetes and DR vs HC. In dominant model, mutant allele showed significant association with DNR and DR vs HC. No significant association of rs1800624 was detected with DR and its sub-groups, non-proliferative DR (NPDR) and proliferative DR (PDR) vs DNR. Dividing NPDR into mild, moderate and severe, heterozygous TA genotype showed significant association with moderate and severe NPDR vs DNR. In DNR and DR groups, TA genotype was significantly associated with raised sRAGE.

**Conclusion:**

rs1800624 RAGE gene polymorphism might be a risk factor for T2DM and NPDR in Pakistani patients. Raised sRAGE levels have a positive correlation with PDR and are associated with heterozygosity of rs1800624 polymorphism in DNR and DR groups

## INTRODUCTION

Diabetic individuals are at an increased risk of developing diabetic retinopathy (DR), the most frequent micro-vascular complication and a principal reason of vision-loss in adults. The prevalence of DR in Pakistan from 1990 to 2017 was 8.6% and vision threatening DR was 28.2%.^1^ Multiple genes have been investigated for their relationship with DR, but the exact mechanism involved in pathogenesis is still ambiguous. Persistent hyperglycaemia activates the advanced glycation end products (AGEs) formation thus contributing to diabetic complications via direct tissue injury, or by initiating specific receptors for AGE (RAGE). Being a part of immunoglobulin superfamily, RAGE gene polymorphisms may influence DR development.^2^

RAGE-mediated signalling triggers transcription factors, thus promoting a pro-coagulant state in the micro capillaries of the retina. Soluble RAGE (sRAGE) is an innately appearing inhibitor of RAGE mediated signalling pathways and may counteract its damaging effects by trapping RAGE ligands.^3^ 374T/A (rs180024) RAGE gene polymorphism, positioned in the promoter region, causes upregulation of the RAGE and plays a fundamental role in the DR pathogenesis. It has been assessed for its association with DR in Asian, Caucasian, Indian, Malaysian and other populations.^2^,^4^,^5^ Literature on the genetic factors involved in pathogenesis of DM and DR in Pakistani patients is very inadequate^6^-^8^ and it is important to explore these factors to provide adequate and prompt therapy.

The present study was planned to determine the relationship of 374T/A (rs1800624) polymorphism in the gene encoding RAGE with Type-2 diabetes mellitus (T2DM), DR and serum sRAGE levels in Pakistani patients.

## METHODS

It was a descriptive case-control study conducted from January 2017 to December 2018, at Pakistan Railway Hospital (PRH), Al-Shifa Eye Hospital Rawalpindi and District Head-quarter Hospital Mirpur. Ethical approval was taken from Institutional review committee of Riphah International University (Appl.# Riphah/ERC/16/0182. August 16, 2016) and written informed consent of the participants was obtained. Non-probability convenient sampling was done. A total of 450 subjects were included:150 diabetics with no retinopathy (DNR), 150 diabetics with retinopathy (DR) and 150 non-diabetic healthy controls (HC). Sample size was estimated with the help of Cochran’s sample size formula.^9^ Inclusion criteria was patients with T2DM for a minimum period of five years, related to three major ethnic groups of Pakistani populations, Punjabi, Pathan and Kashmiri. Exclusion criteria was patients with type1DM, hematologic, malignant, acute / chronic hepatic, cardiovascular, renal diseases, or acute ophthalmic infections. Demographic data regarding age, gender, ethnicity and duration of DM was noted. Ophthalmological examination was carried out by certified ophthalmologists using grading coloured fundus photography. Fasting venous blood sample was obtained for genomic DNA isolation and biochemical analysis of serum sRAGE, FPG, lipid and renal parameters.

Genomic DNA extraction from whole blood was done by 5-7% Chelex (Bio-Rad) and its concentration was measured by Nano-Drop 2000c Spectrophotometer (USA) at 260/280 nm. rs1800624 polymorphism was identified by Tetra-primer ARMS-PCR by using; two common, forward outer and reverse outer primers and two T and A allele specific primers for simultaneous detection of two alleles in a single reaction: (FO-5’-GGGGCAGTTCTCTCCTCACTTGTAAA) and (RO-5’-CCTTTGGGACAAGAGTCCTTCAGG); and two forward & reverse inner allele specific primers, A-allele (FI-5’-GCCTTCATGATGCAGGCCCTAA) and T-allele (RI -5’- AGACTGTTGTCTGCAAGGGTGGAA).^10^

**Fig.1:**
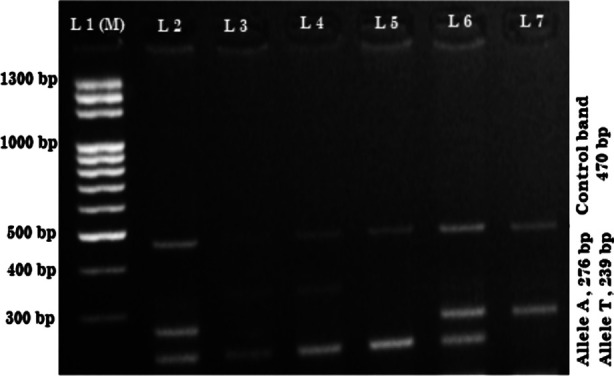
“Electrophoresis pattern of Tetra-primer ARMS-PCR for rs1800624 polymorphism. PCR products resolved on 2% agarose gel. Lane 1: molecular size markers (M) 100 bp; lane 2 and 6: TA; lanes 3-5: TT; lane 7: AA genotypes. T allele 239bp, A allele 276 bp, control band 470 bp.”

PCR was done in thermal cycler, using DreamTaqTM Green PCR master mix (2x), following company’s guidelines. A volume of 20μl was put into a 0.2 mL PCR tube including 2μL template DNA (100 ng/μL); 1μL primer mix 10 picomol/μL each, 8μL master mix and 9μL DNase-free water. PCR was done by DNA denaturation at 95°C (5 min) accompanied by 30 cycles of 95°C (30 sec), annealing at 60°C (30 sec), extension at 72°C (30 sec) and an ultimate extension at 72°C (10 min). Resolution of PCR amplificants was done on two percent agarose gel (5ul ethidium bromide per 100ml agarose soln.) to envision the DNA bands under ultra violet light in Syngene gel imaging system (G:Box, USA). The amplicons derived are shown in Fig.1.

Serum sRAGE was measured by ELISA. Data was analysed by using SPSS version 22.0. We used Fisher’s exact /Chi-square test and odds ratio (OR) for allelic/genotype frequencies and association of genotypes with DM, DR and ethnicity. For comparison of biochemical variables among groups, analysis of variance (ANOVA) with Tukey’s post-hoc test was used. Pearson correlation and regression analysis were done for correlation and risk assessment.

## RESULTS

The mean age of HC, DNR and DR subjects was 55.90 ± 10.90, 58.16 ± 9.42 and 56.25 ± 8.5 years respectively. The male to female ratio was 44.7% vs 55.3% in HC, 47% vs 56% in DWR and 42% vs 58% in DR group respectively. There was a significantly increased percentage of Punjabis in DR group vs HC & DNR groups (p = 0.007 & 0.006 respectively). The total period of DM was significantly more in DR group vs DNR group (13.02 ± 5.71 vs 9.31 ± 4.01 years). Detailed comparison of demographic and biochemical characteristics was done by the authors in a preceding study.^8^

According to chi-square test results, heterozygous TA genotype showed meaningful association with DM and DR vs HC while homozygous TT was taken as reference. Mutant allele was significantly associated with DNR and DR vs HC. In dominant model, minor allele A was association with DNR and DR vs HC. These associations were significant at univariate and multinomial regression analysis (Table-I).

**Table-I:** Association analysis of genotype and allele distribution of 374T/A polymorphism among study groups.

374T/A	HC N=150 N (%)	DNR N=150 N (%)	DR N=150 N (%)	OR (95% CI)	p Value	Regression analysis OR (95% CI)	p-value
***Genotypes***						
TT	139 (92.7)	120 (80)	115(76.7)	Ref 1			
TA	9 (6)	26 (17.3)	29 (19.3)	3.34 (1.50-7.42)^a^ 3.89 (1.77- 8.56)^b^ 1.15 (0.64-2.08)^c^	0.001^a^ 0.0003^b^ 0.610^c^	3.34 (1.50-7.42)^a^ 3.89 (1.77-8.56) ^b^	0.003^a^ 0.001^b^
AA	2 (1.3)	4 (2.7)	6(4)	2.31(0.41-12.87)^a^ 3.62(0.71-18.31)^b^ 1.56 (0.43-5.69)^c^	0.423^a^ 0.149^b^ 0.536^c^		
***Allele***							
T	287 (95.7)	266 (88.7)	259(86.3)				
A	13 (4.3)	34 (11.3)	41 (13.7)	2.82 (1.45-5.46)^a^ 3.49 (1.83-6.66)^b^ 1.23 (0.80-2.01)^c^	0.001 ^a^ <0.0001^b^ 0.386^c^	2.82 (1.45-5.46)^a^ 1.86 (1.35-2.58)^b^	0.002 ^a^ 0.002^b^
***Dominant Model***							
TT TA+AA	139 (92.7) 11 (7.3)	120 (80) 30 (20)	115 (76.7) 35 (23.3)	3.15 (1.51-6.57)^a^ 3.84(1.86-7.91)^b^ 1.21 (0.70-2.11)^c^	0.001 ^a^ 0.00001^b^ 0.483^c^	3.15 (1.51-6.57)^a^ 1.96 (1.36- 2.81)^b^	0.002 ^a^ <0.001^b^
***Recessive Model***							
TT+TA AA	148 (98.7) 2 (1.3)	146(97.3) 4 (2.7)	144(96) 6(4)	2.02(0.36- 11.24)^a^ 3.08(0.61-15.52)^b^ 1.52 (0.42-5.50)^c^	0.684^a^ 0.282^b^ 0.521^c^		

“HC: healthy controls, DNR: diabetic with no retinopathy, DR: diabetic retinopathy, a: HC vs DNR, b: HC vs DR, c: DNR vs DR, OR: odds ratio, CI: confidence interval. p value < 0.05 is considered significant.”

When DR group was split into NPDR and PDR, no notable association of heterozygous and mutant homozygous genotypes among DNR, NPDR and PDR groups was seen (Table-II).

**Table-II:** Association analysis of genotype distribution of 374T/A polymorphism among DNR, NPDR and PDR Groups.

Genotypes	DNR n=150 (%)	NPDR n=100 (%)	PDR n=50(%)	OR (95% CI)	p-value
TT	120 (80.0)	72 (72)	43(86)	Ref	
TA	26 (17.3)	23(23)	6(12)	1.47(0.78 – 2.77)^a^ 0.64(0.24-1.67)^B^	0.226^a^ 0.362^b^
AA	4(2.7)	5(5)	1(2)	2.08(0.54-8.01)^a^ 0.69(0.07-6.41)^B^	0.307^a^ 1^b^

“DNR: diabetic with no retinopathy, NPDR: non - proliferative diabetic retinopathy, PDR: proliferative diabetic retinopathy, a: DNR vs NPDR, b: DNR vs PDR.”

Stratifying NPDR group into mild, moderate and severe, a significant association of TA genotype with moderate and severe NPDR was observed vs DNR group in univariate and multinomial regression analysis (Table-III).

**Table-III:** Association analysis of genotype distribution of 374T/A polymorphism among DNR and NPDR sub-groups.

Genotypes	DNR n=150 (%)	NPDR Mild n=44 (%)	NPDR Moderate n=34 (%)	NPDR severe n=22 (%)	OR (95% CI)	p-value	Regression analysis OR (95% C1)	p-value
TT	120 (80.0)	39(88.6)	21(61.8)	12(54.5)	Ref			
TA	26 (17.3)	3(6.8)	12(35.3)	8(36.4)	0.35(0.10-1.23)^a^ 2.63(1.15-6.02)^b^ 3.07(1.14-8.28)^c^	0.09^a^ 0.01^b^ 0.03^c^	2.63(1.15 - 6.02)^b^ 3.77(1.42-10.03)^c^	0.021^b^ 0.008^c^
AA	4(2.7)	2(4.5)	1(2.9)	2(9.1)	1.53(0.27-8.72)^a^ 1.42(0.15-13.41)^b^ 5(0.82-30.19)^c^	0.638^a^ 1^b^ 0.113^c^		

“DNR: diabetic with no retinopathy, NPDR: non - proliferative diabetic retinopathy, PDR: proliferative diabetic retinopathy, a: DNR vs NPDR Mild, b: DNR vs NPDR moderate, c: DNR vs NPDR severe.”

**Fig.2:**
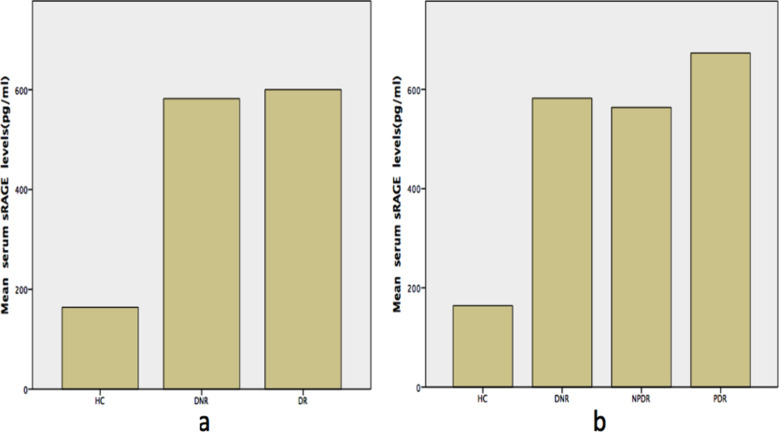
Comparison of mean serum sRAGE levels (pg/ml) among HC, DNR and DR groups (a) and HC, DNR, NPDR and PDR groups (b) “HC, healthy controls; DNR, diabetic without retinopathy; DR, diabetic retinopathy; NPDR, non - proliferative diabetic retinopathy; PDR, proliferative diabetic retinopathy.”

In Punjabi and Kashmiri ethnic groups, combined TA + AA genotype, was significantly associated with DNR and DR vs HC (OR: 2.98, CI: 0.99- 9.00, p= 0.044 and OR: 3.06, CI: 1.07-8.73, p= 0.03). In DNR and DR groups, TA genotype was significantly associated with raised sRAGE in both univariate and multinomial regression analysis (p = 0.001 & p = 0.026) and (p = 0.001 & p= 0.01) respectively (Table-IV). Comparison of serum sRAGE and FPG concentrations among study groups showed significantly elevated serum sRAGE in DR and DNR groups vs HC (600.12 ± 238.54 and 582.04 ± 206.04 vs. 164.05± 70.53 pg/ml) and in PDR group vs NPDR and DNR (745.98 ±180.14, 527.18 ± 231.2 and 582.04 ± 206.04 pg/ml respectively) (Fig.2). Fasting plasma glucose, total cholesterol, triglyceride, LDL-C, urea and creatinine were raised in DNR and DR groups vs HC (p= <0.001each) whereas FPG, total cholesterol and LDL-C were found elevated in DR vs DNR group (p=< 0.001, 0.003 and 0.001 respectively). Serum sRAGE and FPG were positively correlated in DR group (r: 0. 232, p = 0.004). Other associated parameters were studied by the authors previously.

**Table IV:** Association analysis of serum sRAGE levels and other biochemical parameters with 374T/A genotypes in DNR and DR groups.

*DNR GROUP*					

Mean ± SD	TT (n=120)	TA (n=26)	AA (n=4)	p-value	Multinomial Regression p-value
Serum sRAGE (pg/ml)	553.0 ± 207.6	714.3 ± 159.1	592.1 ± 45.9	0.001*	0.001*
FPG (mg/dl)	162.7 ± 50.6	175.5 ± 56.5	164.3 ± 56.7	0.48	
Total cholesterol (mg/dl)	201.8 ± 41.4	212.1 ± 50.4	192.4 ± 40.7	0.54	
HDL-C (mg/dl)	43.5 ± 8.5	44.5 ± 7.4	49.5 ± 5.7	0.21	
LDL-C (mg/dl)	127.6 ± 41.1	136.3 ± 44.7	103.5 ± 42.5	0.19	
Triglyceride (mg/dl)	183.8 ± 65.7	170.7 ± 50.0	170.8 ± 67.5	0.55	
Urea (mg/dl)	30.1 ± 9.1	33.2 ± 10.0	32.5 ± 9.2	0.26	
Creatinine (mg/dl)	1.10 ± 0.38	1.17 ± 0.31	0.96 ± 0.36	0.33	

*DR GROUP*					
	*TT (n=115)*	*TA (n=29)*	*AA (n=6)*	*p-value*	

Serum sRAGE (pg/ml)	569.1 ± 229.6	696.4 ± 255.4	729.7 ± 224.1	0.026*	0.01*
FPG (mg/dl)	192.1 ± 56.2	189.7 ± 55.5	172.8 ± 32.9	0.70	
Total cholesterol (mg/dl)	218.8 ± 40.8	223.8 ± 46.2	244.0 ± 40.2	0.34	
HDL-C (mg/dl)	44.4 ± 8.0	47.6 ± 7.9	44.0 ± 7.0	0.15	
LDL-C (mg/dl)	141.7 ± 36.7	145.1 ± 45.2	168.8 ± 32.2	0.21	
Triglyceride (mg/dl)	176.1 ± 44.1	170.9 ± 40.7	176.6 ± 42.2	0.84	
Urea (mg/dl)	33.2 ± 9.9	32.8 ± 8.8	34.1 ± 6.4	0.94	
Creatinine (mg/dl)	1.17 ± 0.38	1.24 ± 0.38	1.28 ± 0.42	0.58	

“DNR, diabetic with no retinopathy; DR, diabetic retinopathy; sRAGE, soluble RAGE; p value < 0.05 significant;

* TT vs TA.”

## DISCUSSION

We investigated the association of rs1800624 RAGE gene polymorphism with diabetes, diabetic retinopathy and serum sRAGE levels in the Pakistani patients. Our results demonstrated a meaningful association of heterozygous TA genotype and mutant allele variant with diabetes and DR vs HC (p= <0.05 each) in both univariate and multinomial regression analysis (Table-I). We also found a meaningful association of heterozygous genotype with moderate and severe NPDR in comparison to DM group (Table-III). Consistent with our findings, a significant association of rs1800624 with NPDR (p = 0.048) was seen in Type-2 diabetic Indian population.^11^ A notable association of mutant A allele with elevated risk of DR was also found in Caucasian and Asian populations.^4^

In contrast to this, various studies carried out in Asians, Caucasians, Chinese and Malaysians revealed no meaningful association of diabetic retinopathy with rs1800624 polymorphism.^5^,^12^ Few meta-analysis supported the relationship of mutant A allele with protection against DR^13^ while others favoured the association with DR.^4^,^14^ The inconsistent results may be due to difference in genetic make-up and lifestyles among populations. The moderate association of heterozygous TA genotype with moderate and severe NPDR in our patients was not recognised when sub-groups were studied together and need to be deduced very cautiously. The RAGE gene is sited on the chromosome 6p21.3 adjacent to HLA locus where almost thirty polymorphisms have been recognized. Other gene variants in same region may be implicated in pathogenesis of DR. We found a notable association of combined TA + AA genotype with DWR and DR vs HC (P= 0.044 & P= 0.03) in Punjabi and Kashmiri ethnic groups.

We found significantly elevated serum sRAGE in PDR patients. Mutant homozygous genotype had a significant association with raised serum sRAGE in HC, whereas, heterozygous TA genotype had a significant association with raised sRAGE in DNR and DR groups (Table-IV). These findings suggest that raised serum sRAGE and rs1800624 polymorphism may be linked with pathogenesis of T2DM and DR in our population and may serve as a valuable clinical indicator for vascular disease risk assessment. Similar findings were observed by Simó-Servat et al. in a review article in Tunisian and Malaysian populations.^15^ We also observed a statistically significant positive correlation between serum sRAGE and FPG in DR group. To protect tissues from AGE-induced damage, cells process more AGE receptors which facilitate cellular uptake and breakdown of AGEs. Thus, sRAGE has cytoprotective effects against AGEs–RAGE interaction either by confiscating them or contending with RAGE for ligand binding.^3^ Several studies showed significantly declined sRAGE as retinal disease advanced from NPDR to PDR, suggesting increased binding with circulating AGEs and is a useful indicator for individual variations in vulnerability to DR.^3^,^16^,^17^

### Limitations of the study:

Due to a cross-sectional study design we could not ascertain whether the associations were of a causal nature. We investigated 374T/A SNP in RAGE gene promoter region and we could not eliminate the chance that other SNPs in this region might be involved in the progression of DR. Further studies on a larger scale are still needed to assess whether this RAGE gene polymorphism is a predisposing factor for DM and DR risk and to clarify the relationship between rs1800624 polymorphism with serum sRAGE in Pakistani population.

## CONCLUSION

374T/A (rs1800624) RAGE gene polymorphism might be a risk factor for T2DM and NPDR in Pakistani patients. Raised sRAGE concentrations have a positive correlation with PDR and are associated with heterozygosity of rs1800624 polymorphism in diabetics with and without retinopathy.

### Authors Contribution:

**SQ:** conceived, designed and did biochemical analysis, statistical analysis & editing and is responsible and accountable for the accuracy of the work

**MA:** biochemical analysis & editing.

**AKN:** did review and final approval of manuscript.
